# Genital Reconstruction After Weight Loss in Adipose Male Patients: A Case report

**Published:** 2014-04-02

**Authors:** Daniel Robert Arno Sattler, Silke Altmann, Manfred Infanger, Nauras Abuagela, Sarah Maj Schneegans, Hans-Georg Damert, Armin Kraus

**Affiliations:** Department for Plastic, Aesthetic and Handsurgery, University Hospital Magdeburg, Magdeburg, Germany

**Keywords:** obesity, genital reconstruction, pendulous abdomen, abdominoplasty, penis reconstruction

## Abstract

**Objective:** We introduce our surgical technique in two male genital reconstruction cases out of 15 post-bariatric patients. **Methods:** At our Department for Plastic Surgery at the University Hospital Magdeburg, 15 patients, 6 male and 9 female, underwent a surgical abdominoplasty after weight loss in 2009. **Results:** The average weight of the 15 patients was preoperatively 197.2 kg and the average hospital stay was of 14 days. In 2 cases, a second procedure for male genital reconstruction was necessary. After primary dietary measures and weight loss, we performed genital reconstruction in a second step with a sleeve-, Z-, VY-plasty and a “bilobed flap” to restore function and appearance of the male genitalia. In these patients, the average weight was 207.5 kg and hospital stay lasted 32 days. **Conclusion:** The increase of patients with obesity-related genital deformities will be expected in the future. Therefore, more controlled long-term studies should be published to develop guidelines for genital reconstruction techniques in plastic surgery.

Weight gain and subsequent weight loss cause significant alterations in the appearance of the male genitalia. The terms “buried,” “hidden,” and “concealed” penis refer all to maladies in which the functional and visual penile length is obscured. The classification of Metcalfe and Rink^1^ distinguishes a congenitally abnormal fat deposition in the mons pubis area in infancy versus the acquired fat deposition and ptosis in the pubic area in adults. The growth in overweight and obesity rates among adults is a major public health concern. Obesity is a known risk factor for numerous health problems including hypertension, high cholesterol, diabetes, cardiovascular diseases, and cancer. More than half of the adult population in the European Union is overweight and the rate of obesity has reached 16.9%.^2^ According to the Centers for Disease Control and Prevention, the incidence of obesity in the United States increased to 35.7% from 1995 to 2011.^3^ Male patients complain of burying of the penis causing secondary sexual dysfunction, hygiene issues, discomfort, and esthetic concerns. Even with weight loss, most of these deformities persist. Indications for repair are numerous as the concealment can trap urine and make voiding difficult. These patients may present with balanitis, a urinary tract infection, or even urinary retention.^4^ Hygiene is also a concern, in both the infant period and adolescence, as problems holding the penis and the spraying of urine can be significant.

## CASE PRESENTATION

**Figure 1:**
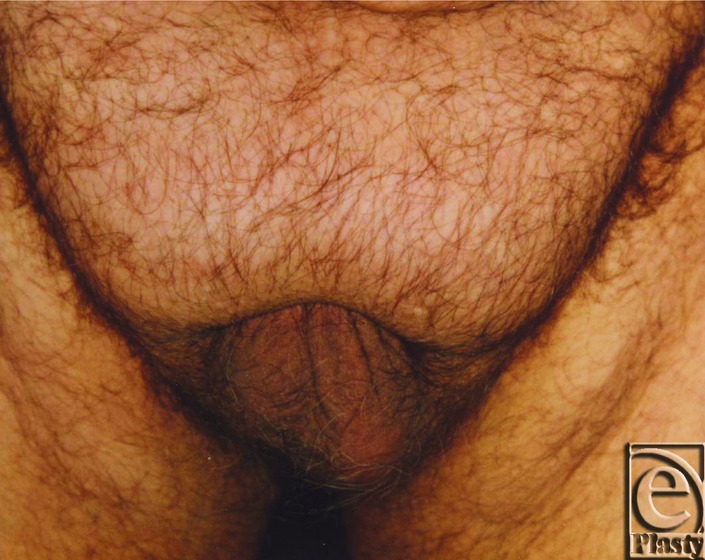
Retracted penis and scrotum.

**Figure 2:**
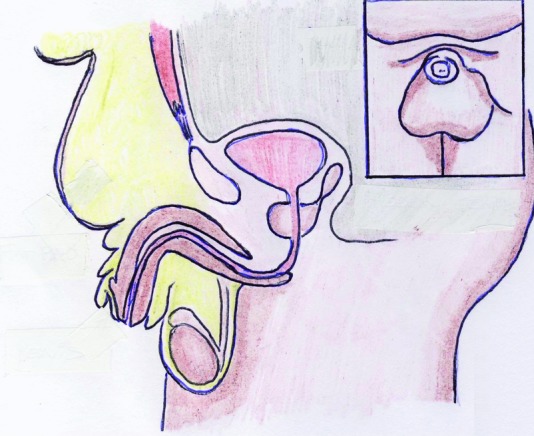
Subcutaneous fat deposition of the mons pubis.

**Figure 3:**
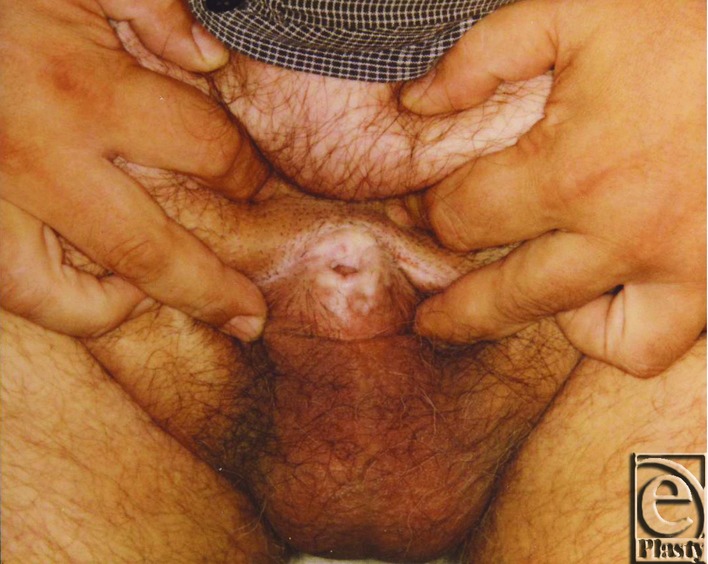
Foreskin phimosis.

At our Department of Plastic Surgery at the University Hospital Magdeburg, 15 patients — 6 male and 9 female — underwent abdominoplasty after weight loss in 2009. The average weight of the 15 patients was preoperatively 197.2 kg and the average hospital stay was 14 days. In 2 male patients, a second procedure was necessary to reconstruct the male genital. In these patients, the average weight was 207.5 kg and hospital stay was 32 days. We introduce a 46-year-old male patient with a body mass index (BMI) of 45 kg/m^2^ (height: 196 cm, weight: 166 kg). He presented with excessive skin and fat in the lower abdomen area and localized subcutaneous fat deposition in the mons pubis area leading to a complete retraction of the penis and scrotum (Figs 1, 2). He complained of not having sexual intercourse for more than 1.5 years. Furthermore, the abdominal skin excess as well as the retraction of his genitals caused serious infections of the glans penis due to neglected hygiene. A protraction of the glans was not possible due to the acquired foreskin phimosis of the penis (Fig 3). Physical examination revealed the following functional complaints:

–Chronic infection of the glans penis and the foreskin
–Fibrotic contracture of the distal part of the foreskin
–Inability to perform sexual intercourse
–Insufficient micturition

## MATERIAL AND METHODS

**Figure 4:**
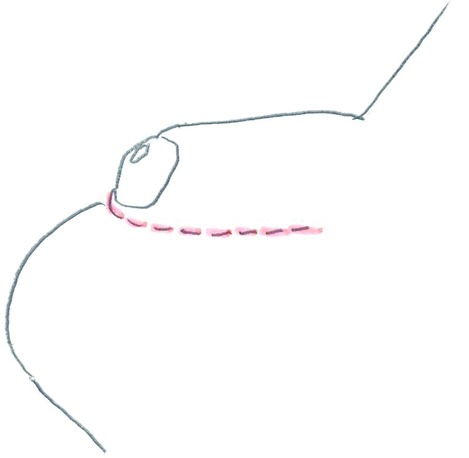
Sleeve-plasty.

We initiated body weight reduction by dietary measures over a period of 6 months as a precondition for the public health security to cover the treatment costs. As a result, our patient reduced his BMI from 45 to 39 (kg/m^2^) and kept his weight of 141 kg constant for more than 6 months. The second step was to perform an abdominoplasty to remove excessive skin and fat from the lower abdomen so that the mons pubis was no longer covered by excessive skin. After the abdominoplasty, skin and soft tissue was able to recover from chronic topic infections and mycosis. The genital reconstruction was done as the third step of our treatment concept. We performed an open lipectomy of the mons pubis area as well as at the scrotum according to the surgical technique described by Horton et al.^5^ After reduction of the subcutaneous deposits, we were able to perform the reconstruction of the penis and scrotum due to the resulting excessive skin in this area after open lipectomy. Contouring of the penile shaft was performed by a sleeve plasty, following a Z-plasty in the area of the penile root to ensure the filling of the corporal bodies (Fig. 6). The fibrous contracture of the penile foreskin was released by VY-plasty. A proportional scrotum was formed using a bilobed flap from the scrotal skin (Fig 4). Similar surgical techniques were described by Alter.^4^^,^^6^

## OUTCOME

The postoperative management was accompanied by recurrent wound healing problems. Therefore, we initiated an antibiotic therapy with second-generation cephalosporin. The performed wound swab revealed a bacterial spectrum of *Escherichia coli*, *Proteus vulgaris*, and *Enterococcus faecalis*. According to the microbiological result, we changed the antibiotic regime to ampicillin and sulbactam. At postoperative day 10, we performed a revision at the dorsal scrotum to excise a secondary healing skin area and close it primarily. On the 21st day, stitches were taken out and the indwelling catheter was removed. The patient was discharged after 32 days (Fig. 7). Lymph edema was treated successfully by manual lymph drainage in our physiotherapy department.

## DISCUSSION

**Figure 5:**
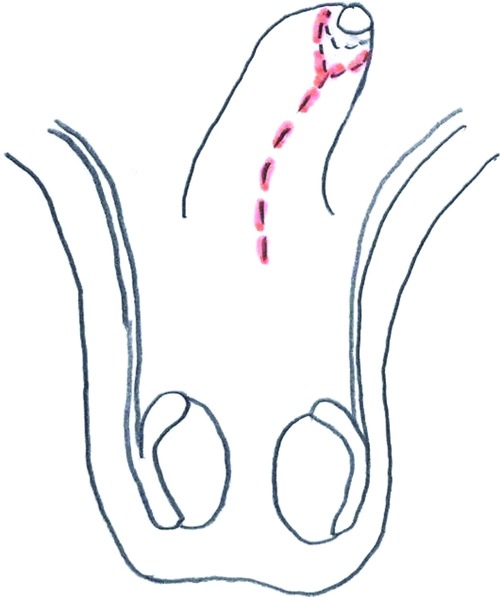
VY-plasty.

**Figure 6:**
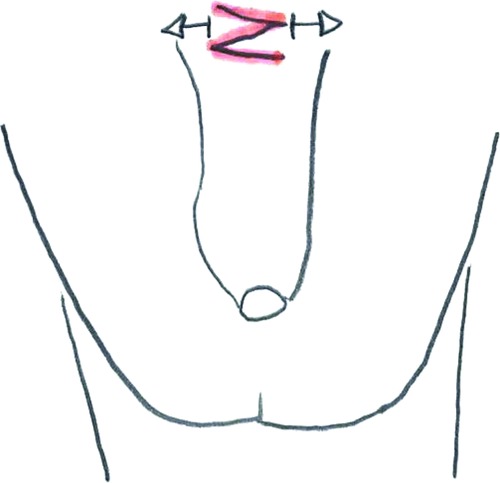
Z-plasty.

**Figure 7:**
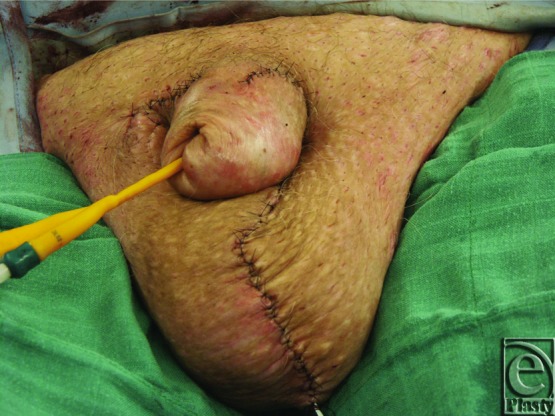
Postoperative result.

Several authors report about the difficulties associated with genital reconstruction after trauma, aphallia, penis retraction, and gender reassignment.^1^^,^^4^^,^^5^^,^^7^^,^^8^ Postoperative occurrence of lymph edema after genital reconstruction was first described by Stark et al. and led to persistent discomfort in our patient.^9^ The few long-term follow-up studies available confirm the difficulty of such surgery in terms of complications and the limits of the final achievable outcomes. Scientific progress in penile reconstruction seems slow, with a lack of controlled studies, high rate of loss to follow-up, and a lack of validated assessment measures.^8^ Beside the congenital reasons for genital retraction, obesity with an increased incidence in our population will become a more important aspect as a reason for acquired dysfunction and retraction of the male genitalia. In our described case, we were able to treat the chronic wound infection and subsequently reconstruct his male genitalia and preserve its functionality successfully (Fig 5). The genital retraction was removed by open lipectomy technique followed by several local skin flaps and plasties with the result of a penis as a formally and esthetically integrated unit and the patient was able to have sexual intercourse. After 12 month of lymph drainage, therapy lymph edema occurrence appeared only after long standing and subsequently stabilized. Deepithelization of the penile shaft and split skin graft coverage is an option. Because of the poor elastic quality, which is essential for erectile function, it is reserved for few indications. Split skin grafts provide a risk of hypertrophic scaring and subsequent contractures.^10^^,^^11^ The questionnaire developed by Borkenhagen et al.^12^ for postoperative patient satisfaction after mamma reduction was modified according to our procedure performed.^12^ The most positive feature of the questionnaire mentioned was return of sexual activity, whereas the most negative feature was the prolonged lymph edema. Weight was effectively reduced by a combination of conservative and surgical procedures. The patient was reevaluated after 2 years and presented with a stable body weight of 145 kg. Chronic burying of the male genitalia with its associated dampness is likely to occur at BMI greater than 30 kg/m^2^ and can lead to severe functional problems. Irritation of the penile skin and glans can lead to balanitis xerotica obliterans, midshaft or foreskin phimosis, and eventual destruction of the penile skin.^4^ Even with weight loss, the buried penis remains because of the ptotic skin, lack of dartos-to-Buck attachments, and chronic inflammation.

## CONCLUSION

The worldwide increase of obese patients with BMI greater than 30 kg/m^2^ has been reported in international studies and literature.^2^^,^^3^ An increase of obese patients with acquired forms of genital deformities will be expected in the future. Therefore, more controlled long-term studies should be published to develop guidelines for genital reconstruction techniques in plastic surgery.^8^
